# Stress, Anxiety and Depressive Symptoms, Burnout and Insomnia Among Greek Nurses One Year After the End of the Pandemic: A Moderated Chain Mediation Model

**DOI:** 10.3390/jcm14041145

**Published:** 2025-02-10

**Authors:** Argyro Pachi, Christos Sikaras, Dimitrios Melas, Sofia Alikanioti, Nikolaos Soultanis, Maria Ivanidou, Ioannis Ilias, Athanasios Tselebis

**Affiliations:** 1 Psychiatric Department, Sotiria Thoracic Diseases Hospital of Athens, 11527 Athens, Greece; irapah67@gmail.com (A.P.); dimelmed@hotmail.com (D.M.); olina.alikanioti@gmail.com (S.A.); soultanisnikolaos@gmail.com (N.S.); mivanidou24@gmail.com (M.I.); 2 Nursing Department, Sotiria Thoracic Diseases Hospital of Athens, 11527 Athens, Greece; cris.sikaras@gmail.com; 3 Department of Endocrinology, Hippocration General Hospital of Athens, 11527 Athens, Greece; iiliasmd@yahoo.com

**Keywords:** stress, anxiety, depressive symptoms, burnout, insomnia, nurses

## Abstract

**Background/Objectives**: Several studies have reported alarming rates of mental health issues and sleep problems among nurses even in the post-pandemic era. The objective was to investigate the prevalence of stress, anxiety and depressive symptoms, burnout and insomnia among nurses in Greece one year after the end of the pandemic and to construct a mediation model evaluating the impact of stress on insomnia, the chain mediating roles of depressive symptoms and burnout, as well as the moderating role of anxiety symptoms in the model. **Methods**: This cross-sectional study was conducted online in July 2024 and included 380 hospital nurses who completed the Depression Anxiety Stress Scale (DASS-21), the Copenhagen Burnout Inventory (CBI) and the Athens Insomnia Scale (AIS). **Results**: The prevalence rates of stress, anxiety and depressive symptoms, burnout and insomnia were 33.9% with 95% confidence interval (CI): [0.292, 0.390], 33.3% (95% CI: [0.284, 0.381]), 35% (95% CI: [0.302, 0.400]), 46.8% (95% CI: [0.399, 0.502]) and 56.1% (95% CI: [0.509, 0.611]), respectively. Multiple regression analysis indicated that the Depression subscale of the DASS-21 explained 40.6% of the variance in the AIS, while an additional 7.6% was explained by the CBI and another 1.3% rate by the Stress subscale of the DASS-21. Mediation analysis revealed that stress affected insomnia both directly and indirectly through the chain mediating roles of depressive symptoms and burnout. Anxiety symptoms moderated the chain mediation path by enhancing the negative impact of stress on depressive symptoms. **Conclusions**: The proposed moderated chain mediation model introduces certain factors influencing insomnia and explains how changes in any one of these factors effectuate changes in the other factors, offering insights for individualized interventions.

## 1. Introduction

The COVID-19 pandemic lasted almost three years, from 30 January 2020 when the World Health Organization (WHO) designated the outbreak as a Public Health Emergency of International Concern (PHEIC) [1] and labeled it a pandemic on 11 March 2020 [2], until 5 May 2023 when the WHO announced its official conclusion [3]. The onset of the pandemic completely overwhelmed the health systems in most countries. Major deficiencies of ventilators, intensive care beds, personal protective equipment and significant shortages of health workers exposed the flawed health policies implemented in previous years. Notably, the pandemic had a profound social, economic and psychological impact on populations worldwide, fundamentally changing daily life and affecting public health, far beyond the direct impact of the virus itself [4,5]. Psychological distress prevailed during the pandemic [6], with reports showing increased rates of insomnia, anxiety and depressive symptoms, stress and burnout among healthcare workers, particularly nurses [7]. Prolonged and close interaction with patients combined with increased exposure to their emotional distress possibly explains the particular vulnerability of nurses [8].

Sleep disturbances among nurses during the pandemic have understandably drawn plenty of attention from researchers, as they can contribute to physical and mental health problems and are associated with an increased risk of workplace errors, burnout and job change [9,10,11,12]. Insomnia, shift work causing circadian rhythm disruption and insufficient sleep are common among nurses, especially hospital nurses who almost exclusively engage in shift work [13,14]. During the pandemic, nurses were constantly faced with stressful situations while navigating through complicated procedures of care and treatment. Chronic exposure to increased amounts of stress often causes insomnia [15], whereas good-quality sleep protects us against the negative impact of stress. Studies during the pandemic reported that insomnia in nurses was associated with stress, anxiety, depressive symptoms and burnout [16,17,18,19,20]. Results from prior research suggested that depressive symptoms and anxiety comorbidity may have a negative impact on insomnia severity [21], and according to a longitudinal study, nurses who experienced burnout symptoms early in their professional life reported more insomnia problems at follow-up [22]. Also, a recent cross-sectional study identified anxiety, depressive symptoms and burnout as risk factors for sleep disturbance among clinical nurses [23], and another study reported that job burnout was a risk factor contributing to insomnia among nurses with long COVID-19 [24].

According to the 11th revision of the International Classification of Diseases (ICD-11), the code QD85 is attributed to burnout syndrome, which is considered to result from ongoing work-related stress that has not been effectively addressed and resolved [25]. The main component of this syndrome is the feeling of exhaustion experienced by professionals in the occupational context, which is significantly related to job dissatisfaction [26,27]. Healthcare professionals were among the first individuals in the workforce in whom burnout syndrome was investigated [28]. All studies over the past few decades consistently reported high levels of burnout in nursing staff [29,30,31,32,33] and simultaneously evidenced constantly high associations of burnout with depressive symptoms and anxiety [34,35].

The significant correlation between burnout and depression symptoms was established from the first publications examining the nature of burnout [36]. However, it should be noted that typical depression symptoms such as low self-esteem, feelings of guilt, hopelessness and suicidal tendencies are not typical symptoms of burnout [37]. On the other hand, in burnout, extended removal from work on vacation has beneficial effects, whereas a depressive episode necessitates psychotherapeutic intervention and/or medication. The existing literature converges on the view that burnout and depression symptoms are separate entities [38,39], and research studies suggest that depressive symptoms are among significant factors influencing burnout levels of nursing staff [40,41].

Stress is implicated in the etiology of a major depressive episode, and people who report chronic stress are more likely to be diagnosed with a depressive disorder [42,43]. By definition, burnout is understood as the outcome of prolonged unresolved occupational stress [44]. Employment conditions and workload predict anxiety and perceived stress among employees, and lack of administrative support is the most important factor responsible for the increase in anxiety [45]. According to research, anxiety and depression symptoms are important factors affecting burnout in nurses [35]. Moreover, scholars have investigated the correlation between insomnia and workplace stressors [46]. Consequently, nurses who work in stressful and demanding settings are at increased risk for insomnia, anxiety, depressive symptoms and burnout [47].

Numerous studies have documented the prevalence of these aforementioned mental health issues among nurses [48,49], but few have focused on their interrelations [50,51,52]. Also, past and current studies after the official conclusion of the pandemic reveal that these psychological effects could persist for a long time [53,54,55,56,57,58,59,60]. Therefore, in this study, we aim to assess the levels and explore the interrelations between stress, anxiety and depressive symptoms, burnout and insomnia among nurses working in Greek hospitals one year after the end of the pandemic. Furthermore, a literature review did not retrieve previous research investigating the chain mediating effects of depressive symptoms and burnout as well as the moderating role of anxiety symptoms in the linkage between stress and insomnia. The conceptual framework for this study was that stress would influence insomnia both directly and indirectly through the chain mediating role of depressive symptoms and burnout. Moreover, the presence of anxiety symptoms would regulate the association between stress and depressive symptoms and ultimately aggravate insomnia. The above hypothetical schema underlies the main purpose of this study which was to unravel the underlying pathways through which stress affects insomnia and identify significant factors influencing insomnia, thus offering guidance for individualized interventions to alleviate insomnia symptoms. To address this objective, we formulated the following hypotheses:

**Hypothesis 1.**  *Stress is positively associated with and predicts insomnia.*

**Hypothesis 2.**  *Depressive symptoms mediate the relationship between stress and insomnia.*

**Hypothesis 3.**  *Burnout mediates the relationship between stress and insomnia.*

**Hypothesis 4.**  *Depressive symptoms and burnout play a chain mediating role in the relationship between stress and insomnia.*

**Hypothesis 5.**  *Anxiety symptoms moderate the influence of stress on depressive symptoms and the strength of the association between stress and depressive symptoms increases with the rise in the levels of anxiety symptoms. Concurrently, anxiety symptoms regulate the indirect link from stress to insomnia through their influence on depressive symptoms.*

## 2. Materials and Methods

### 2.1. Research Design

To address the above objectives, we conducted a cross-sectional study using a homogeneous convenience sampling method [61]. Recruited participants were nurses working in Greek hospitals with a minimum of one year of professional experience. The data were collected via Google Forms, and the online questionnaire was shared electronically through the email addresses retrieved from scientific and professional registries of Greek nurses. The invitation email delivered to participants included an anonymous link that provided access to the Google Forms online research platform. Consenting participants declared that they agree to participate voluntarily by marking the “I agree” option as stated on the first page of the online questionnaire, which was considered informed consent. The sample for this study included nurses who consented to take part and subsequently filled out the other sections of the online questionnaire. The exclusion criteria were hospital nurses who were not on duty for any reason and/or have not worked fulltime for the past month and during the survey period. Also, this study excluded nurses who served in outpatient and non-hospital settings, as well as in formal leadership and advanced practice roles.

### 2.2. Study Participants

The study was conducted in July 2024. Cochran’s formula [62,63] was employed to calculate the sample size. Given that the target population was 27,103 individuals [64], and with a confidence level of 95% (meaning that the z score was 1.96), a confidence interval of 5% (which means that the margin of error for proportion being estimated was 0.05), and an assumption of a 50% response rate (meaning that the population proportion was 0.5), a minimum sample size of 379 participants was required. A total of 500 invitations were emailed, with 380 responses received (response rate: 76%).

### 2.3. Ethical Considerations

This study was conducted following ethical principles outlined in the General Data Protection Regulation (GDPR–2016/679) of the European Union, the World Medical Association Declaration of Helsinki (1975, revised 2008), and the guidelines of the International Committee of Medical Journal Editors. The study protocol was approved by the Ethics Committee of Clinical Research of the General Hospital for Thoracic Diseases of Athens “SOTIRIA” (Approval Number: 20649/16-05-2023).

### 2.4. Measurement Tools

After giving their consent and prior to answering the questionnaires, respondents were asked to provide demographic and professional data, including their gender, age, and years of work experience. Then, they were prompted to fill out the following set of questionnaires.

#### 2.4.1. Depression Anxiety Stress Scale (DASS-21)

The Depression Anxiety Stress Scale-21 (DASS-21) [65] is a measurement tool which comprises three self-report subscales intended to evaluate stress, anxiety and depressive symptoms. Seven statements on a four-point Likert scale make up each subscale ranging from 0 (did not apply to me at all) to 3 (applied to me very much or most of the time). Scores must be multiplied by 2 to determine the final score. Respondents were asked to score the degree to which they have experienced each state over the past week. Elevated scores reflect the increasing experience of stress, anxiety and depressive symptoms. The depressive symptoms subscale evaluates dysphoria, anhedonia, inertia, hopelessness, feelings of sadness, loss of interest or pleasure, self-deprecation and worthlessness (e.g., “I couldn’t seem to experience any positive feeling at all”). The anxiety symptoms subscale evaluates skeletal muscle effects, situational anxiety, autonomic arousal, and the subjective sensation of anxious affect (e.g., “I felt I was close to panic”). The stress subscale estimates non-specific arousal levels such as restlessness, nervousness, excitability, agitation, irritability, overreaction and impatience (e.g., “I felt that I was using a lot of nervous energy”). Different cut-off values exist for the conventional severity levels for each subscale; i.e., scores above 9 are indicative of depressive symptoms, above 7 of anxiety symptoms, and above 14 of stress. The total score is an indicator of general psychological distress. The DASS-21 is a screening tool designed to identify areas of concern and not a diagnostic instrument for assigning patients to specific diagnostic categories proposed in classification systems. The DASS-21 is acknowledged for its robust psychometric properties [66]. In this research, the Greek version of the scale was used [67], and for the present sample, Cronbach’s alpha values of the depression, anxiety and stress subscales were 0.912, 0.902 and 0.914, respectively. The average variance extracted (AVE) value of the DASS-21 depression subscale was 0.582. The square root of this AVE (0.76289) was utilized to check the discriminant validity of this construct against the burnout inventory according to the Fronell–Larcker criterion [68], which dictates that the square root of the average variance extracted by a construct must be greater than the correlation between the construct and any other construct.

#### 2.4.2. Copenhagen Burnout Inventory (CBI)

The Copenhagen Burnout Inventory (CBI) is the most common instrument for burnout assessment [69], consisting of nineteen questions and evaluating the concept of burnout in three subdimensions: the first six questions reflect personal burnout; the next seven questions evaluate work-related burnout; and the last six questions appraise patient-related burnout. The personal burnout subscale measures the level of perceived physical and psychological burnout. Burnout symptoms related to work are evaluated by the work-related burnout subscale. The patient-related burnout subscale assesses perceived physical and psychological burnout from interaction with patients. Response options are rated on a five-point Likert scale. All burnout subscales have scores between 0 and 100, with greater scores indicating increased degrees of occupational burnout. In this study, the Greek version of the CBI was used, which is a valid scale, possessing robust psychometric properties [70]. Cronbach’s alpha coefficient for the entire scale in this study was α = 0.933. A total score of ≥50 [71,72,73,74,75,76,77] indicates professional burnout. The AVE value of the general scale was 0.5199. The square root of this AVE (0.72104) was utilized to check the discriminant validity of this construct against the DASS-21 depression subscale according to the Fronell–Larcker criterion. Also, discriminant validity was established with the scale score’s disattenuated correlations [68], by using the following formula: $rXY÷\sqrt{rXX*rYY}$, where rXY is the correlation between the DASS-21 depression subscale and the burnout inventory, rXX is the reliability of the DASS-21 depression subscale and rYY is the reliability of the burnout inventory. In this study, the score is 0.61 which is below 0.70, suggesting that discriminant validity likely exists between the Copenhagen Burnout Inventory and the DASS-21 depression subscale [68].

#### 2.4.3. Athens Insomnia Scale (AIS)

The Athens Insomnia Scale (AIS) is a self-report measurement tool intended to measure the severity of insomnia (how severely certain sleep difficulties have affected the responders during the past month) using diagnostic criteria set forth by the 10th Revision of the International Classification of Diseases and Related Health Problems (ICD-10). The scale comprises eight items amongst which the first five assess nocturnal sleep (sleep induction, night-time awakenings, final awakening, total sleep duration, and overall sleep quality), and the final three items are related to daytime dysfunction (well-being, functioning, and drowsiness throughout daytime). Response scores for each item range from 0 to 3, and total score spans from 0 to 24, with greater scores signifying increased severity of insomnia. A diagnostic threshold set at 6 indicates insomnia [78]. The AIS is a widely used tool for assessing insomnia. The Greek version of the AIS has demonstrated good psychometric properties [79]. In this study, Cronbach’s alpha coefficient was measured at α = 0.878.

### 2.5. Statistical Analysis

Firstly, since self-report questionnaires were used to collect the data, the Harman single-factor test was employed to examine the common method bias [80]. This technique uses exploratory factor analysis, where all variables are loaded on a single factor and restricted so that there is no rotation in order to determine the proportion of variance explained by the first largest factor. Subsequently, descriptive statistical methods were applied to estimate means and standard deviations for continuous variables and to calculate the proportion of responders that scored above the cutoff values of clinically significant stress, anxiety and depressive symptoms, burnout and insomnia. Furthermore, using *t*-tests and *χ*^2^ tests, we compared the sample to the general population of nurses in Greece regarding years of professional experience, age, and gender, in order to examine the representativeness of the sample. Independent *t*-tests were conducted to assess continuous variables according to gender. Correlations between all variables included in the present study were investigated using Pearson’s correlation test. Linear regression analysis was utilized to determine if the correlated variables were significant predictors of insomnia. Before proceeding with the regression analysis, the prerequisite assumptions were checked, specifically normality by visual examination of the predicted probability plots, linearity through the visual review of scatter plot pairs, and homoscedasticity through residuals’ scatter plot. The independence of residuals was assessed with the Durbin–Watson test. The Variance Inflation Factor (VIF) analysis was performed to determine the absence of multicollinearity in the data. To examine the chain mediation effect of depressive symptoms and burnout between stress and insomnia, we conducted the serial mediation analysis using Hayes’ SPSS Process Macro Model 6 [81]. The moderating role of anxiety symptoms in the chain mediation model was tested using Hayes’ SPSS Process Macro Model 83 [82]. Regression coefficients reported were unstandardized and standardized with their confidence intervals. The 95% confidence intervals were assessed using 5000 bootstrap samples. Finally, analysis of simple slopes was performed to report the regulating effect at different levels of anxiety symptoms. The data analyses were conducted using SPSS software (Version 24.0). For all statistical analyses, statistical significance was set at *p* < 0.05 (two-tailed).

## 3. Results

Since the data acquired for the study were derived from self-reports, common method bias testing was required, and for this purpose, the Harman single-factor method test was utilized. Results from the exploratory factor analysis indicated that the first common factor had an explanation rate of 39.686 percent, which was less than the critical value of 50 percent, suggesting no significant common method bias in this study.

A total of 380 nurses (74 males and 306 females) participated in the study. As for gender, age, and years of professional experience, no significant differences were identified between the study sample and the total population of nurses working in Greece [64]. Overall, 56.1% of the nurses exhibited insomnia symptoms (AIS ≥ 6), 46.8% presented signs of burnout (CBI ≥ 50), while 31.1% experienced symptoms of general psychological distress (DASS-21 > 32) [83]. Regarding the severity levels of stress, anxiety, and depressive symptoms, 33.9%, 33.3% and 35% of the participants scored above the recommended normal values in DASS-21, whereas percentages of 25%, 26.8% and 23.9% were found to have moderate to extremely severe levels of stress, anxiety, and depressive symptoms, respectively. Table 1 presents the mean values and standard deviations of the study variables.

Regarding gender, female nurses evidenced higher mean scores in the DASS-21 total and the stress subscale compared to male nurses (*t*-test *p* < 0.05, 29.53 ± 27.42 vs. 22.67 ± 21.88 and *t*-test *p* < 0.05, 13.47 ± 10.26 vs. 10.67 ± 8.2, Table 1). Additionally, female nurses showed higher scores in the Copenhagen Burnout Inventory (*t*-test *p* < 0.05, 49.64 ± 19.03 vs. 44.91 ± 17.93, Table 1).

AIS correlated positively with DASS-21 total and its three subscales and negatively with work experience. CBI scores were positively associated with both the AIS scores and the DASS-21 total and its subscales. Specifically, the correlation between the Depression subscale of DASS-21 and the CBI was significant (r = 0.563, SE = 0.0352, 95% CI: [0.4941, 0.6319]), indicating a moderate strength of association with large effect size. The Anxiety subscale of the DASS-21 correlated negatively with age and work experience, and moreover, the Depression subscale showed a negative correlation with work experience. As expected, the three DASS-21 subscales positively correlated with each other (Pearson Correlations *p* < 0.01, Table 2).

We ensured that the prerequisites for the regression analysis were satisfied by checking the necessary assumptions in advance. Independence of residuals was tested using the Durbin–Watson test, with a value of 1.843 (Table 3), supporting the absence of autocorrelation. The VIF values of less than 4 indicated a lack of multicollinearity (Table 3). Normality was verified by visually observing the predicted probability plots. Homoscedasticity was explored through visual review of the scatter plot of standardized and predicted residual values. Linearity was confirmed by visually inspecting scatter plots of variable pairs.

We conducted a multiple regression analysis using the stepwise method to explore which factors best explain the scores of the Athens Insomnia Scale (AIS). In the multiple regression analysis, AIS was set as the dependent variable, while age, gender, years of work experience, the Copenhagen Burnout Inventory (CBI), and the subscales of the Depression Anxiety Stress Scale (DASS-21) were set as independent variables. The analysis showed that the Depression subscale of the DASS-21 explained 40.6% of the variance in AIS, while an additional 7.6% was explained by CBI and another 1.3% was explained by the Stress subscale of the DASS-21 (Table 3). The other variables did not contribute significantly to the AIS variance.

**Figure 1:**
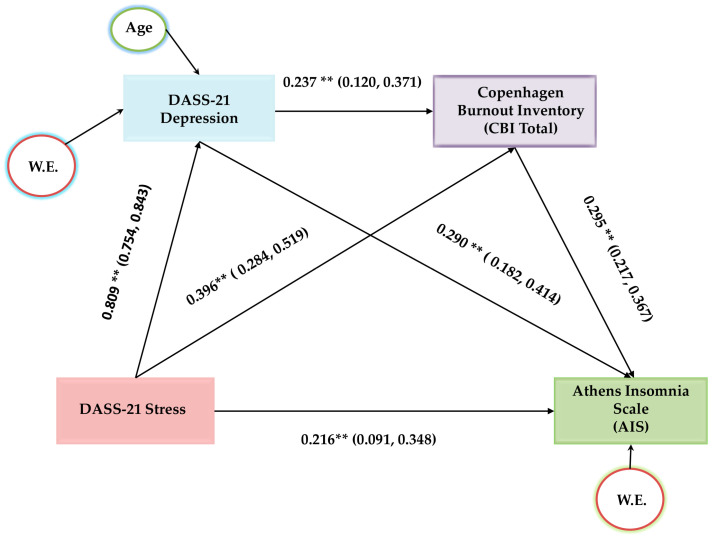
Chain mediation model of depressive symptoms and burnout in the relationship between stress and insomnia. Note: ** *p* < 0.01.

Next, we explored the hypothesis that depressive symptoms and burnout might act as mediators in the relationship between stress and insomnia. In this analysis, the Stress subscale of the DASS-21 was set as the predictor variable, the Depression subscale of the DASS-21 and CBI as the mediator variables and AIS as the outcome variable. Covariates included work experience and age. Hayes’ SPSS Process Macro Model 6 was employed to investigate the chain mediating effect of depressive symptoms and burnout in the relationship between stress and insomnia. The analysis was based on 5000 bootstrap samples. Standardized coefficients for the variables with their confidence intervals are illustrated in Figure 1.

The chain mediation analysis revealed that depressive symptoms and burnout serially mediate the relationship between stress and insomnia. In this context, the covariates, age, and work experience exhibited statistically significant relationships (Figure 1 and Table 4). The total indirect effect of depressive symptoms and burnout on insomnia was found to be statistically significant [b = 0.1932, 95% C.I. (0.1311, 0.1655)]. Furthermore, the direct effect of stress on insomnia in the presence of the mediators was proved significant as well (b = 0.1073, *p* < 0.01). Therefore, there is partial serial mediation of depressive symptoms and burnout on the relationship between stress and insomnia. This model explains 64.3% of the variance in the AIS outcome variable. In particular, the following three pathways yielded indirect effects that contributed to the total mediating effect: (a) DASS-21 Stress subscale → DASS-21 Depression subscale → AIS, which represents 36.3% of the total effect; (b) DASS-21 Stress subscale → CBI → AIS, rendering 18.8% of the total effect; and (c) DASS-21 Stress subscale → DASS-21 Depression subscale → CBI→ AIS, constituting 9.18% of the total effect.

**Figure 2:**
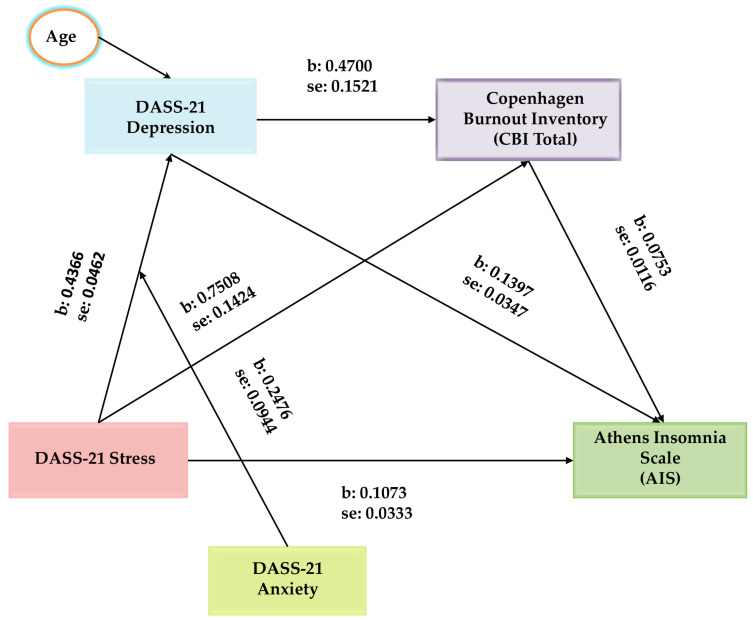
The moderated chain mediation model in this study: stress impacts insomnia through the chain mediation effect of depressive symptoms; and burnout and anxiety symptoms moderate the relationship between stress and depressive symptoms in the first and third paths of the chain mediation.

Lastly, we investigated the process by which anxiety symptoms may moderate the relationship between stress and depressive symptoms. Specifically, we aimed to investigate if the DASS-21 Anxiety subscale acting as a moderator is altering the potency of the indirect effect of the above chain mediation, by means of testing the moderating role of anxiety symptoms in the pathways of the DASS-21 Stress → DASS-21 Depression → AIS and the DASS-21 Stress → DASS-21 Depression → CBI → AIS. To perform this moderation analysis, we utilized the PROCESS method, model 83 (Figure 2).

In both pathways, the index of moderated mediation was significant: b = 0.0009, 95% percentile CI [0.0001, 0.0019], and b = 0.0002, 95% percentile CI [0.0000, 0.0005], providing support for a moderated mediation. A change of 0.37% in depressive symptoms can be attributed to the interaction term. The results revealed a significantly positive moderating role of anxiety symptoms on the linkage between stress and depressive symptoms (b = 0.066, *t* = 2.3841, *p* = 0.0176), Table 5. This shows that at higher levels of anxiety symptoms, the impact of stress on depressive symptoms is reinforced.

**Figure 3:**
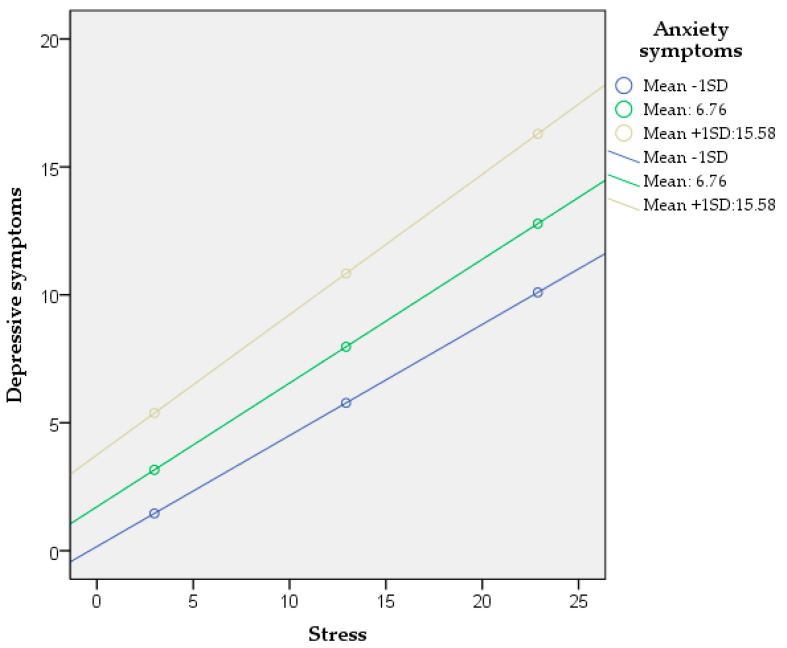
Simple slopes indicating the interaction of the moderation analysis.

Furthermore, to demonstrate how anxiety symptoms moderated the association between stress and depressive symptoms, a simple slope test was performed. High and low levels of anxiety symptoms (plus or minus a standard deviation) were grouped to generate the simple effect analysis diagram (Figure 3). The findings indicated that stress has a significant impact on depressive symptoms in both high and low levels of anxiety symptoms. Nevertheless, compared to nurses with a low level of anxiety symptoms (simple slope = 0.4366, *t* = 9.4585, *p* < 0.001), stress has a stronger predictive effect on depressive symptoms in nurses with a high level of anxiety symptoms (simple slope = 0.5398, *t* = 9.6773, *p* < 0.001). Hence, the impact of stress on depressive symptoms is much stronger at high levels of anxiety symptoms.

## 4. Discussion

Results from this study evidenced a high prevalence of stress, anxiety and depressive symptoms, burnout and insomnia among Greek hospital nurses, one year after the end of the pandemic. According to a meta-analysis of studies published up until March 2021, due to the pandemic, 40% of health workers experienced acute stress, 42% anxiety symptoms, 33% depressive symptoms, 37% burnout and 42% insomnia [84]. A review of studies published until 2021 on mental health outcomes among nurses working in emergency hospital settings reported a rate of 29.55% for moderate to severe symptoms of anxiety, 38.79% for depressive symptoms and 40.66% for insomnia [85]. Meanwhile, in Greece, in a study conducted from mid-November to mid-December 2021, 39.7% among hospital nurses exhibited depressive symptoms, 60.1% scored above the cut-off on state anxiety and 46.8% on trait anxiety [86]. Regarding burnout levels, a study performed in February 2021 indicated that 42.9% of nurses had scores suggestive of burnout [31]. Also, a study conducted in May 2020 among Greek hospital nurses reported a 49.7% prevalence of insomnia and 50.3% of stress [16], whereas in a study conducted approximately two years after the onset of the pandemic [17] and another study conducted two months after the pandemic ended, 61.4% among Greek hospital nurses presented with symptoms of insomnia [87]. Results from studies performed in other countries among healthcare workers after the pandemic are inconclusive, either reporting alarming findings comparable to those during the pandemic [60] or presenting overall improvements in mental health and sleep problems [56]. In Greece, the psychological implications and sleep disturbances seem to persist for a longer period, even after the pandemic [53]. This difference may be attributed to the reduced perceived organizational support and the limited resources of nursing personnel in the Greek National Health System [88,89], who work hard under challenging conditions but are underpaid compared to their colleagues from other OECD countries [90,91].

Among demographic and work-related factors influencing psychological and sleep symptoms, age, gender and work experience were recorded in this study. In agreement with most other studies [92,93,94,95,96] that clearly show that female nurses are the most vulnerable subgroup among healthcare workers, especially regarding the mental health impacts, female nurses in this study reported higher levels of stress and burnout and exhibited higher scores in the DASS-21 compared to their male counterparts. The female gender predominates in the nursing sector, unlike in other working environments, and the literature suggests that women are more likely to be at risk for psychological distress [97,98,99]. Moreover, hospital nurses are particularly prone to experiencing higher levels of stress and burnout than the other non-hospital nurses [100,101]. Also, in this study, age and work experience proved to be among influencing factors for presenting psychological issues and sleep problems. Specifically, age correlated negatively with anxiety symptoms, and work experience was negatively associated with anxiety and depressive symptoms and insomnia. Most of the existing literature justifies these results [102,103,104,105,106], but certain studies argued that the more experienced nurses were also the more anxious ones, possibly because they were assigned to more challenging work tasks [107].

This study focused on the effects of stress on insomnia and further investigated the separate and the chain mediating role of depressive symptoms and burnout and verified the moderating role of anxiety symptoms in nurses working in Greek hospitals one year after the end of the pandemic. The results elucidate the underlying mechanism through which stress affects insomnia and identify significant factors influencing insomnia, thus providing guidance for targeted interventions for nurses. This section includes the major findings which are discussed in conjunction with the other relevant literature.

### 4.1. The Influence of Stress on Insomnia

The results from both the regression and the mediation analysis indicated that stress positively predicted insomnia, thus supporting the first hypothesis. From a neurobiological perspective, sleep/circadian rhythmicity and the stress response system engage the same neural networks [108]. Stress dysregulation involving the sympathetic–adrenomedullary and the hypothalamic–pituitary–adrenocortical systems may lead to insomnia. The literature suggests that stressful experience is the most common precipitating factor of insomnia [109], and work is one of the most common sources of environmental stress [110]. The relationship between occupational stress and insomnia has been investigated by numerous studies, and a recent meta-analysis identified the strength of this association [111]. Research indicates that nurses are a vulnerable and high-risk population for occupational stress, which is a major risk factor for insomnia in nurses [112]. Notably, hospital nurses compared to community and other non-hospital nurses report higher levels of stress [113]. These stressful experiences are related to the nature of the nursing profession and therefore cannot be easily modified, but the identification of the mediating variables between stress and insomnia could offer valuable insights in order to alleviate the effect of stress on insomnia through the effective manipulation of these mediating variables.

### 4.2. The Mediating Role of Depressive Symptoms

The results from the regression analysis indicated that depressive symptoms explained 40.6% of the variance in insomnia, and the mediation analysis revealed that stress was positively related to depressive symptoms that consequently were associated with insomnia, thus supporting the second hypothesis. In this case, depressive symptoms operate as a catalyst between stress and insomnia. Recent research confirms the mediating effect of depressive symptoms in the association between perceived stress and sleep quality among healthcare workers [114]. The experience of stressful negative life events is implicated in the vulnerability to depressive symptomatology [115]. People are more prone to developing negative cognitive–emotional appraisals when they experience increased levels of stress [116]. Similarly, stressful life events may fuel rumination in some individuals, usually combined with negative emotions [117]. In the long run, failure to regulate these negative cognitions and alleviate these emotions would lead to depressive symptoms [118,119]. Furthermore, emotional hyperarousal derived from depressive emotions as a result of perceived stress may disrupt the normal sleep cycle, leading to a spectrum of sleep issues [120,121,122].

### 4.3. The Mediating Role of Burnout

The mediation analysis revealed that burnout was not only an outcome of stress but also played a mediating role between stress and insomnia, thus verifying the third hypothesis. Furthermore, the regression analysis evidenced that burnout explained 7.6% of the variance in insomnia. The literature points to a bidirectional association between burnout and insomnia, suggesting that either might be a risk factor for the other [123,124]. Specifically, stress, burnout and insomnia are reciprocally related in a vicious cycle [124,125]. Dysregulation involving the sympathetic nervous system and/or the hypothalamic–pituitary–adrenal axis is observed in both burnout and insomnia [125]. A prospective study supported that burnout at baseline not only intensified insomnia symptoms over time for individuals already exhibiting these symptoms at baseline, but it was also related to the emergence of new cases at follow-up [126]. A recent longitudinal population-based cohort study indicated that burnout was the strongest among several risk factors for insomnia [127]. A possible mechanism explaining the way that burnout may cause insomnia implicates emotional exhaustion which contributes to increased sleep reactivity and, in turn, leads to hyperarousal before sleep and ultimately to sleep problems, like insomnia [128]. Also, a meta-analysis confirmed the relationship between burnout and sleep disorders in nurses and provided information about influencing variables, such as gender, shift work and workplace violence [129].

### 4.4. The Chain Mediating Role of Depressive Symptoms and Burnout

This study evidenced that depressive symptoms and burnout play a chain mediating role between stress and insomnia, hence confirming the fourth hypothesis. Studies suggest a reciprocal relationship between depression symptoms and burnout, and some researchers identified burnout as a significant predictor of depressive symptoms [130,131,132,133]. Differently, other researchers supported that depressive symptoms can increase the possibility of burnout [127,134], and an earlier study indicated that current depressive symptoms predicted burnout and, furthermore, an underlying susceptibility for depression symptoms as inferred from a personal and familial history of depressive episodes increased the risk for burnout [135]. Also, there is a debate among researchers over the possibility that burnout and depression symptoms overlap [136,137]. In this sense, both depressive symptoms and burnout can be caused by stressors in the workplace [138], and employees in occupational environments that are particularly demanding and stressful, such as healthcare settings, are prone to suffering from comorbid burnout and depressive symptoms [139]. This has led some scholars to reconceptualize burnout symptoms as a form of ‘occupational depression symptoms’ [140]. However, a recent meta-analysis revealed that depressive symptoms and burnout are different and robust constructs with no overlap between them [39]. This meta-analysis stated that concerning the burnout–depression-symptoms relationship, the findings from studies suggest that the effect size of their association is not strong enough to imply that they are the same construct. Moreover, the studies that used the Maslach Burnout Inventory reported lower effect sizes compared to studies that used other measurement tools, and also, the cross-sectional studies reported higher effect sizes compared to the longitudinal ones. In accordance with this notion, in our study, depressive symptoms and burnout were treated as separate constructs since the discriminant validity of the DASS-21 Depression subscale which measures the depressive symptoms and the Copenhagen Burnout Inventory which evaluates burnout was confirmed (in Materials and Methods and Results sections). However, despite being regarded as distinct nosological entities, depression symptoms and burnout syndrome have been found to be intimately linked in a number of studies [141,142]. Furthermore, associations between sleep quality and depressive and burnout symptoms are well established [143,144]. Results from a longitudinal study confirmed that depressive and burnout symptomatology predicted impaired sleep quality, whereas impaired sleep quality did not predict burnout, but only depressive symptoms. The authors argued that work-related stressors also need to be present to elicit an increase in burnout symptoms [143].

### 4.5. The Moderating Role of Anxiety Symptoms

Another important result from this study is that anxiety symptoms moderate the relationship between stress and depressive symptoms, thus confirming the fifth hypothesis. This finding suggests that the degree to which stress and depressive symptoms impact insomnia varies depending on the levels of anxiety symptoms. Stress and anxiety are frequently comorbid, and research has identified the underlying neurobiological mechanism implicated in their bidirectional association [145,146]. Stressful life events often precede anxiety symptoms [147], and the positive association between any kind of anxiety and the severity of insomnia is apparent since shared psychopathological mechanisms, such as emotional overactivity, can be identified [148]. A study exploring the symptom level associations between insomnia, anxiety and depressive symptoms identified uncontrollable worrying and trouble relaxing as the most central symptoms [149]. The role of emotion dysregulation in insomnia has long been proposed [150], and the cognitive model of insomnia describes one of the patterns of subjective experiences of emotions characterized by increased negatively valenced pre-sleep cognitive activity [151,152]. Rumination and worry are the two components of intrusive thoughts [153], and while rumination is related to dysphoric mood and primarily focuses on the causes of this mood state, worry is associated with anxious mood and involves catastrophizing about future stressful events [154]. Another prospective study demonstrated that anxiety and depressive symptoms at baseline predicted insomnia at follow-up [155]. Available evidence indicates a reciprocal relationship between anxiety and depressive symptoms as related to insomnia [156], but the nature of this relationship may differ across specific insomnia symptoms [157,158].

The moderated chain mediation model constructed in this study clarifies the associations among stress, anxiety and depressive symptoms, burnout and insomnia, aiming to provide practical implications for prevention and intervention purposes. The hypothesized mechanisms of links between the aforementioned factors influencing insomnia should raise the possibility that some of these nurses suffering from insomnia may simultaneously display anxiety, depressive symptoms and burnout, all caused by the harmful effects of stress [127,159]. Thus, nurses who present with insomnia symptoms should also be assessed and treated for these other symptoms, and certain risk factors may differentiate nurses who exhibit distinct combinations of these symptoms, which, unless recognized and addressed, would contribute to the chronicity of the disturbance [160]. Although the treatment of choice for chronic insomnia is cognitive–behavioral therapy [161], distinguishing among different constellations of co-occurring symptoms would lead to more focused treatment plans targeting diverse behavioral, psychosomatic, and biological patterns [162,163].

The results from this study highlight the needs for primary prevention measures in the working environment, both at organizational and administrative levels, in order to support nurses in dealing with the stressful working conditions. Early identification of risk factors such as demanding working environment, quality of the hospital, night shifts, not having a permanent job, working experience, excessive workload and inadequate rewards would enable the implementation of effective workplace interventions to prevent or reduce mental health problems among nurses. Targeted interventions such as stress management programs, mindfulness-based interventions, training in positive coping strategies to combat stress, frustration, and emotion management through self-compassion techniques and self-care programs can effectively reduce anxiety and depressive symptoms, burnout and insomnia and may also prove beneficial in managing stress and prevent the occurrence and/or worsening of the above stress-related psychological symptoms among nurses [164,165,166,167].

Certain limitations should be recognized. First, the cross-sectional method of this study precluded inferences about causal associations among the variables, which could only be established with longitudinal studies. Secondly, the employed convenience sampling methodology could hinder the generalizability of the results. Third, data relied on nurses’ self-report measurements conferring a potential self-report bias. Specifically, the Harman single-factor test which was utilized in this study to examine the common method bias has been criticized for showing limited effectiveness in detecting the presence of common method effects, compared to other post hoc tests [168]. Fourth, an important limitation is the lack of information about other work-related variables, namely, nurses’ work department, work shifts, rotation and staff shortages. Finally, this study focused on specific stress-related risk factors influencing insomnia among nurses, with a limited number of covariates. Future studies may consider including more risk as well as protective factors to improve our understanding about their complex interrelations beyond the pandemic, for prevention and intervention purposes.

## 5. Conclusions

High prevalence rates of stress, anxiety and depressive symptoms, burnout and insomnia were observed in hospital nurses one year after the end of the pandemic. More than one in three nurses experienced stress, anxiety and depressive symptoms, almost half of them displayed signs of burnout, and more than half complained about insomnia symptoms. The moderated chain mediation model constructed in this study indicates that stress exerts significant direct and indirect effects on insomnia mediated by depressive symptoms and burnout, whereas anxiety symptoms regulate the relationship between stress and depressive symptoms. These findings provide insights for designing interventions to reduce the adverse effects of stress and insomnia among nurses, with complementary measures focused on improving potential co-occurring anxiety, depressive symptoms and burnout.

## Figures and Tables

**Table 1:** Descriptive statistics of participants.

Gender		Age	Work Experience (in Years)	Athens Insomnia Scale	Copenhagen Burnout Inventory	Depression Anxiety Stress Scale			
						DASS-21 Total	Stress Subscale	Anxiety Subscale	Depression Subscale
Male	Mean	47.57 *	21.89	6.35	44.91 *	22.67 *	10.67 *	5.05	6.94
	N	74	74	74	74	74	74	74	74
	S.D.	10.85	11.92	4.23	17.93	21.88	8.2	7.52	7.99
Female	Mean	44.58 *	19.92	7.31	49.64 *	29.53 *	13.47 *	7.17	8.88
	N	306	306	306	306	306	306	306	306
	S.D.	10.41	11.47	4.92	19.03	27.42	10.26	9.06	9.67
Total	Mean	45.16	20.30	7.12	48.72	28.2	12.93	6.75	8.5
	N	380	380	380	380	380	380	380	380
	S.D.	10.55	11.57	4.80	18.89	26.54	9.95	8.81	9.39

* *t*-test *p* < 0.05.

**Table 2:** Correlations among age, work experience, AIS, CBI and DASS-21 and its subscales.

Pearson Correlation N: 380		Age	Work Experience (in Years)	AIS	CBI	DASS-21 Total	DASS-21 (Stress Subscale)	DASS-21 (Anxiety Subscale)	DASS-21 (Depression Subscale)
Work experience (in years)	r	0.894 **							
	*p*	0.001							
Athens Insomnia Scale (AIS)	r	−0.064	−0.126 *						
	*p*	0.214	0.014						
Copenhagen Burnout Inventory (CBI)	r	−0.031	−0.058	0.587 **	0.72104				
	*p*	0.552	0.257	0.001					
Depression Anxiety Stress Scale (DASS-21 Total)	r	−0.072	−0.132 *	0.662 **	0.586 **				
	*p*	0.161	0.010	0.001	0.001				
DASS-21 (Stress subscale)	r	−0.051	−0.089	0.633 **	0.590 **	0.949 **			
	*p*	0.323	0.083	0.001	0.001	0.001			
DASS-21 (Anxiety subscale)	r	−0.123 *	−0.186 **	0.600 **	0.499 **	0.939 **	0.840 **		
	*p*	0.016	0.000	0.001	0.001	0.001	0.001		
DASS-21 (Depression subscale)	r	−0.034	−0.104 *	0.637 **	0.563 **	0.940 **	0.835 **	0.822 **	0.76289
	*p*	0.508	0.044	0.001	0.001	0.001	0.001	0.001	
AVE (Average Variance Extracted)					0.5199				0.582

* Pearson correlations *p* < 0.05, ** Pearson correlations *p* < 0.01. Note: The square roots of the AVE of the CBI and the DASS-21 Depression subscale are placed on the diagonal.

**Table 3:** Stepwise multiple regression.

Dependent Variable: Athens Insomnia Scale	*R* Square	*R* Square Change	Beta	*t*	*p*	VIF	Durbin–Watson
DASS-21 (Depression subscale)	0.406	0.406	0.290	4.310	0.001 *	3.382	1.843
Copenhagen Burnout Inventory (CBI)	0.483	0.076	0.296	6.438	0.001 *	1.573	
DASS-21 (Stress subscale)	0.496	0.013	0.217	3.143	0.002 *	3.545	

Notes: Beta = standardized regression coefficient; * Correlations are statistically significant at the *p* < 0.01 level (only statistically significant variables are included).

**Table 4:** Chain mediation analysis of depressive symptoms and burnout on stress/insomnia relationship.

Variable	b	SE	*t*	*p*	95% Confidence Interval	
					LLCI	ULCI
DASS-21 Stress → DASS-21 Depression	0.7809	0.0267	29.2765	0.0000	0.7284	0.8333
DASS-21 Stress → CBI	0.7508	0.1424	5.2712	0.0000	0.4708	1.0309
DASS-21 Depression → CBI	0.4700	0.1521	3.0906	0.0021	0.1710	0.7690
DASS-21 Stress → AIS	0.1073	0.0333	3.2235	0.0014	0.0418	0.1727
DASS-21 Depression → AIS	0.1397	0.0347	4.0234	0.0001	0.0714	0.2079
CBI → AIS	0.0753	0.0116	6.4684	0.0000	0.0524	0.0982
^(1)^ DASS-21 Stress → DASS-21 Depression → AIS	0.1091	0.0290	3.7620		0.0513	0.1655
^(2)^ DASS-21 Stress →CBI → AIS	0.0565	0.0146	3.8698		0.0306	0.0885
^(3)^ DASS-21 Stress→ DASS-21 Depression → CBI →AIS	0.0276	0.0091	3.0329		0.0109	0.0464
Covariates						
Age → DASS-21 Depression	0.1540	0.0560	2.7505	0.0062	0.0439	0.2641
W.E. → DASS-21 Depression	−0.1498	0.0512	−2.9254	0.0036	−0.2505	−0.0491
W.E. → AIS	−0.0853	0.0369	−2.3117	0.0213	−0.1578	−0.0127
Effects						
Direct	0.1073	0.0333	3.2235	0.0014	0.0418	0.1727
* Total Indirect	0.1932	0.0309			0.1311	0.1655
Total	0.3005	0.0192	15.6336	0.0000	0.2627	0.3383

Notes: W.E., work experience (in years); Ind1: ^(1)^ DASS-21 Stress → DASS-21 Depression → AIS = DASS-21 Stress → DASS-21 Depression × DASS-21 Depression → AIS; Ind2: ^(2)^ DASS-21 Stress → CBI → AIS = DASS-21 Stress → CBI × CBI → AIS; Ind3: ^(3)^ DASS-21 Stress → DASS-21 Depression → CBI → AIS = DASS-21 Stress → DASS-21 Depression × DASS-21 Depression → CBI × CBI → AIS. * Total Indirect = Ind1 + Ind2 + Ind3, based on 5000 bootstrap samples.

**Table 5:** Moderated chain mediation analysis of the effect of the DASS-21 Anxiety on the association between the DASS-21 Stress and the Athens Insomnia Scale (AIS) through the DASS-21 Depression.

Direct Relationships						
Variable	b	SE	*t*	*p*	95% Confidence Interval	
					LLCI	ULCI
DASS-21 Stress → DASS-21 Depression	0.4366	0.0462	9.4585	0.0000	0.3459	0.5274
DASS-21 Anxiety → DASS-21 Depression	0.2476	0.0944	2.6214	0.0091	0.0616	0.4333
DASS-21 Stress × DASS-21 Anxiety → DASS-21 Depression	0.0066	0.0028	2.3841	0.0176	0.0012	0.0121
DASS-21 Stress → CBI	0.7508	0.1424	5.2712	0.0000	0.4708	1.0309
DASS-21 Depression → CBI	0.4700	0.1521	3.0906	0.0021	0.1710	0.7690
DASS-21 Stress → AIS	0.1073	0.0333	3.2235	0.0014	0.0418	0.1727
DASS-21 Depression → AIS	0.1397	0.0347	4.0234	0.0001	0.0714	0.2079
CBI → AIS	0.0753	0.0116	6.4684	0.0000	0.0524	0.0982
Covariates						
Age → DASS-21 Depression	0.1038	0.0516	2.0101	0.0451	0.0023	0.2053
Effects						
Direct	0.1073	0.0333	3.2235	0.0014	0.0418	0.1727
**Moderated Indirect Relationships**						
Indirect 1: DASS-21 Stress → DASS-21 Depression → AIS						
DASS-21 Anxiety (mean − 1SD)	0.0610	0.0173	3.5260		0.0288	0.0972
DASS-21 Anxiety (mean)	0.0672	0.0188	3.5744		0.0322	0.1062
DASS-21 Anxiety (mean + 1SD)	0.0754	0.0213	3.5399		0.0359	0.1189
Index of Moderated Mediation	0.0009	0.0005			0.0001	0.0019
Indirect 2: DASS-21 Stress → CBI → AIS	0.0565	0.0147	3.8435		0.0309	0.0886
Indirect 3: DASS-21 Stress→ DASS-21 Depression → CBI → AIS						
DASS-21 Anxiety (mean − 1SD)	0.0155	0.0053	2.9245		0.0059	0.0266
DASS-21 Anxiety (mean)	0.0170	0.0058	2.9310		0.0066	0.0293
DASS-21 Anxiety (mean + 1SD)	0.0191	0.0066	2.8939		0.0073	0.0333
Index of Moderated Mediation	0.0002	0.0001			0.0000	0.0005

## Data Availability

The data that support the findings of this study are available from the corresponding author [A.T.], upon reasonable request.
